# Effect of serum autoantibodies on the COVID-19 patient’s prognosis

**DOI:** 10.3389/fmicb.2023.1259960

**Published:** 2023-11-30

**Authors:** Weiming Zhang, Yue Tao, Yijia Zhu, Qisi Zheng, Fenghua Hu, Wenbo Zhu, Jian Wang, Mingzhe Ning

**Affiliations:** ^1^Department of Laboratory Medicine, Nanjing Drum Tower Hospital, The Affiliated Hospital of Nanjing University Medical School, Nanjing, Jiangsu, China; ^2^Department of Infectious Diseases, Nanjing Drum Tower Hospital, The Affiliated Hospital of Nanjing University Medical School, Nanjing, Jiangsu, China

**Keywords:** autoimmunity, autoantibody, ANA, ENA, coronavirus disease 2019, prognostic marker

## Abstract

**Objectives:**

Virus infection closely associated with autoimmune disease. The study aimed to explore the autoantibody profiles and the correlation of autoantibodies with the disease severity and the prognosis of the coronavirus disease 2019 (COVID-19) patients.

**Methods:**

Three hundred thirty-seven hospitalized COVID-19 patients from 6th to 23rd January 2023 were enrolled. Logistic and Cox regression analyses were used to analyze the risk factors for the patient’s disease severity and outcome. The association between Anti-extractable nuclear antigen antibody (ENA) positivity and the prognosis of COVID-19 patients was analyzed using Kaplan–Meier survival curves.

**Results:**

137 of COVID-19 patients were detected positive for antinuclear antibody (ANA), 61 had positive results for ENA, and 38 were positive for ANA and ENA. ANA positivity rate was higher in non-severe illness group (*p* = 0.032). COVID-19 patients who died during hospitalization had a high rate of ENA positivity than convalescent patients (*p* = 0.002). Multivariate logistic regression showed that ANA positivity was a protective factor for the disease severity of COVID-19. Multivariate Cox regression analysis revealed that ENA positivity, white blood cells count (WBC), aspartate aminotransferase (AST), Creatinine (CREA), and CRP were independent risk factors for the outcome of COVID-19 patients, and that COVID-19 patients with ENA positivity had a lower cumulative survival rate (*p* = 0.002).

**Conclusion:**

A spectrum of autoantibodies were expressed in COVID-19 patients, among which ANA and ENA positivity was associated with the severity and prognosis of COVID-19. Therefore, autoantibodies may help to assess the disease severity and prognosis of COVID-19 patients.

## 1 Introduction

Although vaccinations have been applied around the world, the severe acute respiratory syndrome coronavirus-2 (SARS-CoV-2) continues its effects (Viruses, 2020; Zhou P. et al., 2020). The COVID-19 caused by the SARS-CoV-2 has posed a significant financial and medical burden on humans. According to data from the World Health Organization (WHO), to data, there have been more than 0.76 billion confirmed cases of COVID-19, including about 6.8 million deaths globally (Wong et al., 2023). Bilateral interstitial pneumonia was the predominant cause of death from COVID-19 (Schaller et al., 2020). Besides, patients with COVID-19 have an imbalance of the immune system, which is primarily characterized by cytokine storm syndrome, hyperactivation of immune cells, and dysregulated initiated and acquired immunity (Hu et al., 2021; Luo et al., 2021; Woodruff et al., 2022).

Virus infection including Epstein–Barr virus (EBV), cytomegalovirus (CMV), human immunodeficiency virus (HIV), and human T lymphotropic virus 1 (HTLV-1) has an established association with several autoimmune diseases (Gugliesi et al., 2021; Jakhmola et al., 2021; Quaglia et al., 2021). Genetic susceptibility and hyperactivation of the host immune system may trigger autoimmune diseases (Esmaeili et al., 2017; Scappaticcio et al., 2021). Similar to patients infected with other viruses, infected with SARS-CoV-2 show altered autoantibody profiles (Zhou Y. et al., 2020; Peker et al., 2021; Scappaticcio et al., 2021b), such as, antinuclear antibodies, antithyroid antibodies, chromatin proteins, ribosomal proteins, and immune-related proteins (Bastard et al., 2020; Chang et al., 2021; Ulndreaj et al., 2022). These autoantibodies are closely correlated with the disease severity and the prognosis of COVID-19 patients and may trigger long-COVID-19 symptoms (Pascolini et al., 2021; Stjepanovic et al., 2022; Son et al., 2023).

The present study retrospectively analyzed the expression of many autoantibodies in hospitalized COVID-19 patients to evaluate the association between autoantibodies and disease severity and clarify the role of autoantibodies in the prognosis of COVID-19 patients.

## 2 Methods

### 2.1 Study participants

A total of 337 COVID-19 patients who were admitted to Nanjing Drum Tower Hospital, Affiliated Hospital of Medical School, Nanjing University from 3rd to 19th January 2023 were enrolled in this study. According to the Diagnosis and Treatment Plan for Novel Coronavirus Infection (Trial version 10) issued by China’s National Health Commission, the diagnostic criteria of COVID-19 were as follows: (1) clinical manifestations of SARS-CoV-2 infection; (2) a positive result on nuclear acid or antigen of SARS-CoV-2. The clinical classification of COVID-19 was as follows: (1) mild COVID-19 mainly manifested as upper respiratory tract infection, including dry and sore throat, cough, and fever. (2) moderate COVID-19 manifested as persistent high fever for more than 3 days or (and) cough, polypnea, but the respiratory rate (RR) was less than 30 times per minute and the oxygen saturation over 93% during inspiratory breathing at rest. Imaging shows the characteristic manifestations of COVID-19 pneumonia. (3) severe COVID-19 adults had any of the following symptoms that could not be explain by other reasons other than SARS-CoV-2 infection: (i) present of polypnea that RR ≥30 per minute; (ii) oxygen saturation ≤ 93% during inspiratory breathing at rest; (iii) arterial partial pressure of oxygen (PaO_2_)/the fraction of inspired oxygen (FiO_2_) ≤ 300 mmHg (1 mmHg = 0.133 kPa); (iv) progressive exacerbation of clinical symptoms, pulmonary imaging showed that the focus of infection progressed significantly >50% within 24–48 h. (4) critical patients exhibited one of the following conditions: (i) respiratory failure is present and mechanical ventilation is required; (ii) shock occurs; (iii) combined with other organ failure and requires intensive care. In the present study, 178 patients with mild or moderate COVID-19 were classified into the non-severe illness group, and 159 patients with severe or critical COVID-19 were classified into the severe illness group. The exclusion criteria were as follows: concurrent with autoimmune diseases. Written informed consent was obtained from each patient. The study protocol was approved by Ethics Committee of Nanjing Drum Tower Hospital (20222-746).

### 2.2 Data collection

Data were gathered on the patient’s general medical history, clinical symptoms, contact history, chronic medical history, radiological imaging, laboratory measurements, treatments, complications, and outcomes. The timelines included the onset time of COVID-19, admission time, exacerbation time [occurrence of acute respiratory distress syndrome (ARDS), respiratory failure or death], and outcome or discharge time. Disease-free survival was defined as the time from the first symptom onset to the occurrence of ARDS, respiratory failure, or death.

### 2.3 ANA indirect immunofluorescence (IIF) testing and evaluation

ANA determination was performed using the indirect immunofluorescence (IIF) method with the HEp-20-10 liver biochip (Monkey) (Euroimmune AG, Luebeck, Germany) kit at a dilution of 1:100 according to the manufacturer’s recommendation in the collected samples. The evaluation was performed by a laboratory specialist using a fluorescence microscope (Eurostar III plus). The fluorescence intensity of the positive control was assumed as 4+, so the titer intensity values were evaluated as ±(borderline), 1+ to 4+ at ×400 objective. In this process, an evaluation was performed considering International Consensus on ANA Patterns (ICAP) standards (Damoiseaux et al., 2016).

### 2.4 ENA

Serum extractable nuclear antigens (ENA) were detected by a line immunoassay method using the Euroline ANA-profile 1 (IgG) kit (Euroimmun AG, Luebeck, Germany). Each strip consisted of nRNP/Sm (U1-nRNP), Sm, SS-A, recombinant Ro52 (Ro-52, 52 kDa), SS-B, DNA topoisomerase I (Scl-70), PM-Scl, histidyl-tRNA synthetase (Jo-1), centromere protein B (CENP B), dsDNA, nucleosome, histone, and pyruvate dehydrogenase complex antigens and was assayed according to the manufacturer’s protocol.

### 2.5 IL-6 detection

Serum IL-6 levels were detected by Vazyme QD-S2000 using a commercial kit (Vazyme Medical Technology, Nanjing, China). All operations were performed in accordance with the kit instructions.

### 2.6 Detection of serum autoantibodies

Serum was used for autoantibodies detecting in COVID-19 patients. ANAs were detected using indirect immunofluorescence assay on HEp-2 cells (Euroimmun, Lübeck, Germany, CF191019AA). ENAs spectrum was measured by immunoblot (Euroimmun, Lübeck, Germany, D191004AE). ANAs positivity is defined as one or more positive ANAs fluorescence or antibody spectrum tests.

### 2.7 Statistical analysis

Data were analyzed using the SPSS version 15.0 (SPSS Inc., Chicago, IL, the United States) and R software (version 4.1.3, R Foundation, Vienna, Austria). Continuous variables were presented as the median and interquartile range (IQR), and categorical variables were expressed as numbers and percentages. Continuous variables between two groups were compared using the t-test (normal distribution) or Mann–Whitney U test (nonnormal distribution). Categorical variables were compared using the Chi-square test. Survival analysis was performed using the Kaplan–Meier method, and the difference was assessed by a two-tailed log-rank test. A *p* < 0.05 was considered statistically significant.

## 3 Results

### 3.1 Demographics and clinical characteristics of COVID-19 patients

A total of 337 COVID-19 patients were enrolled in this study with a median age of 78 years old (range 67–87 years old), including 120 females and 217 males. Among them, 159 patients were classified into the severe illness group, and 178 patients were classified into the non-severe illness group. The median days of hospitalization were 11 (6–16) days. A total of 46 patients died of COVID-19 during the hospitalization, and 291 patients recovered from COVID-19 and discharged (Table 1).

**Table 1:** Demographics and clinical characteristics of COVID-19 patients.

Variables	Patients (*n* = 337)	ANA		*p*-value	ENA		*p*-value
		Positive (*n* = 137)	Negative (*n* = 200)		Positive (*n* = 61)	Negative (*n* = 276)	
Age median, IQ (range), years	78.0 (67.0, 87.0)	80.0 (68.0, 87.0)	77.0 (65.8, 86.3)	0.149	78.0 (67.0, 87.0)	78.0 (66.8, 87.0)	0.835
Male	217	83	134	0.247	30	187	**0.006**
Hypertension	166	57	109	**0.025**	26	140	0.263
Diabetes	102	33	69	**0.041**	16	86	0.448
Malignancy	51	19	32	0.592	6	45	0.202
WBC median, IQ (range), 1 × 10^9^/L	6.9 (5.0, 9.8)	7.2 (5.0, 9.8)	6.7 (5.0, 9.8)	0.590	7.3 (4.9, 10.4)	6.7 (5.0, 9.7)	0.751
NEU median, IQ (range), 1 × 10^9^/L	5.4 (3.5, 8.2)	5.5 (3.4, 8.2)	5.2 (3.6, 8.2)	0.746	5.7 (3.3, 8.9)	5.2 (3.6, 7.8)	0.796
LYM median, IQ (range), 1 × 10^9^/L	0.9 (0.6, 1.3)	1.0 (0.6, 1.3)	0.9 (0.6, 1.3)	0.348	0.8 (0.6, 1.3)	0.9 (0.7, 1.4)	0.131
HB median, IQ (range), g/L	117.0 (101.0, 130.0)	114.0 (98.3, 124.0)	120.5 (102.0, 133.0)	**0.005**	116.0 (102.0, 128.0)	117.0 (101.0, 131.0)	0.904
PLT median, IQ (range), 1 × 10^9^/L	203.0 (144.0, 273.0)	213.5 (146.3, 290.8)	194.5 (142.5, 257.3)	0.086	178.0 (144.0, 260.0)	206.5 (144.0, 273.3)	0.489
ALT median, IQ (range), U/L	18.6 (12.1, 31.9)	18.6 (12.2, 27.4)	18.7 (12.1, 33.3)	0.489	20.0 (13.1, 29.3)	18.6 (11.7, 32.3)	0.912
AST median, IQ (range), U/L	21.3 (16.1, 31.1)	22.0 (15.8, 29.9)	20.6 (16.2, 32.1)	0.913	22.5 (15.8, 32.0)	21.0 (16.3, 31.1)	0.632
TBA median, IQ (range), μmol/L	3.3 (2.0, 5.6)	3.6 (1.8, 6.3)	3.2 (2.0, 5.2)	0.762	3.9 (1.9, 6.3)	3.3 (2.0, 5.5)	0.608
LDH median, IQ (range), U/L	264.0 (216.0, 354.0)	255.0 (214.0, 354.0)	266.5 (218.3, 354.0)	0.832	260.0 (238.0, 394.0)	264.0 (212.3, 350.5)	0.375
CREA median, IQ (range), μmol/L	67.0 (55.0, 91.0)	67.0 (55.0, 91.0)	67.0 (54.0, 90.3)	0.984	62.0 (52.0, 83.0)	69.0 (55.0, 92.1)	0.059
PT median, IQ (range), second	11.9 (11.2, 12.6)	11.8 (11.2, 12.7)	11.9 (11.3, 12.5)	0.942	11.9 (11.3, 12.6)	11.8 (11.2, 12.6)	0.885
CK median, IQ (range), U/L	47.0 (30.0, 88.0)	44.0 (30.0, 75.5)	51.5 (30.3, 93.3)	0.401	45.0 (24.0, 88.0)	47.0 (30.0, 86.8)	0.583
CKMB median, IQ (range), U/L	12.0 (9.0, 15.0)	11.0 (9.0, 14.0)	12.0 (10.0, 15.0)	0.105	12.0 (9.0, 14.0)	12.0 (9.0, 15.0)	0.722
TNT median, IQ (range), μg/L	0.019 (0.011,0.037)	0.018 (0.011,0.041)	0.020 (0.011,0.035)	0.636	0.018 (0.012,0.032)	0.019 (0.010,0.039)	0.966
CRP median, IQ (range), mg/L	30.7 (5.6, 66.3)	30.9 (6.0, 65.8)	30.4 (5.5, 66.3)	0.812	43.7 (10.0, 68.9)	29.0 (5.2, 65.8)	0.160
PCT median, IQ (range), ng/mL	0.064 (0.034,0.155)	0.059 (0.034,0.134)	0.068 (0.034,0.178)	0.540	0.056 (0.035,0.134)	0.065 (0.033,0.161)	0.856
IL-6 median, IQ (range), ng/L	14.3 (3.7, 49.0)	11.4 (3.4, 45.2)	17.9 (4.1, 50.8)	0.197	21.6 (6.1, 76.0)	12.0 (3.3, 42.3)	**0.039**
Days of Hospitalization median (min,max), day	11.0 (1.0, 54.0)	10.0 (1.0, 54.0)	12.0 (1.0, 53.0)	0.384	11.0 (1.0, 44.0)	11.0 (1.0, 54.0)	0.937
Severe illness	159	55	104	**0.032**	33	126	0.232
Death	46	18	28	0.821	16	30	**0.002**

ALT, alanine aminotransferase; ANA, antinuclear antibody; AST, aspartate aminotransferase; CK, creatine Kinase; CKMB, creatine kinase-MB; CREA, creatinine; CRP, c-reactive protein; ENA, anti-extractable nuclear antigen antibody; HB, hemoglobin; IL-6, interleukin 6; LDH, lactate dehydrogenase; LYM, lymphocyte; NEU, neutrophile; PCT, procalcitonin; PLT, platelet; PT, prothrombin time; TBA, total bile acid; TNT, troponin T; WBC, white blood cell.

ANA positivity was detected in 41% (137/337) of COVID-19 patients, ENA positivity was detected in 18% (61/337), and 11% (38/337) COVID-19 patients were positive for both ANA and ENA. Those involved patients were grouped according to ANA or ENA positivity. As comparison, the proportion of COVID-19 patients with hypertension, diabetes was higher in ANA-negative patients than in ANA-positive patients. Besides, hemoglobin was higher in ANA-negative patients, which may be related to impaired hemoglobin biosynthesis caused by SARS-CoV-2 (Kronstein-Wiedemann et al., 2022). Serum IL-6 level was higher in ENA-positive COVID-19 patients than in ENA-negative COVID-19 patients. However, other clinical features were comparable between ANA-positive and ANA-negative groups or ENA-positive and ENA-negative groups (Table 1). In those patients with ANA positivity, the most detected ANA pattern was Nuclear fine/coarse speckled (31%) followed by Nuclear fine/coarse speckled and Cytoplasmic dense fine/ fine speckled (17%). Nuclear Homogeneous account for 11%, Homogeneous/Clumpy/Punctate nucleolar and Cytoplasmic dense fine/ fine speckled all account for 9% (Supplementary Table S1). In anti-ENA autoantibodies, the most detected antibody was anti-RO-52 (25%), followed by anti-CB (15%), anti-M2 antibody accounting for 10%, and anti-Scl-70 antibody accounting for 7% (Supplementary Table S2).

### 3.2 The proportion of ANA and ENA positivity in different groups

**Figure 1:**
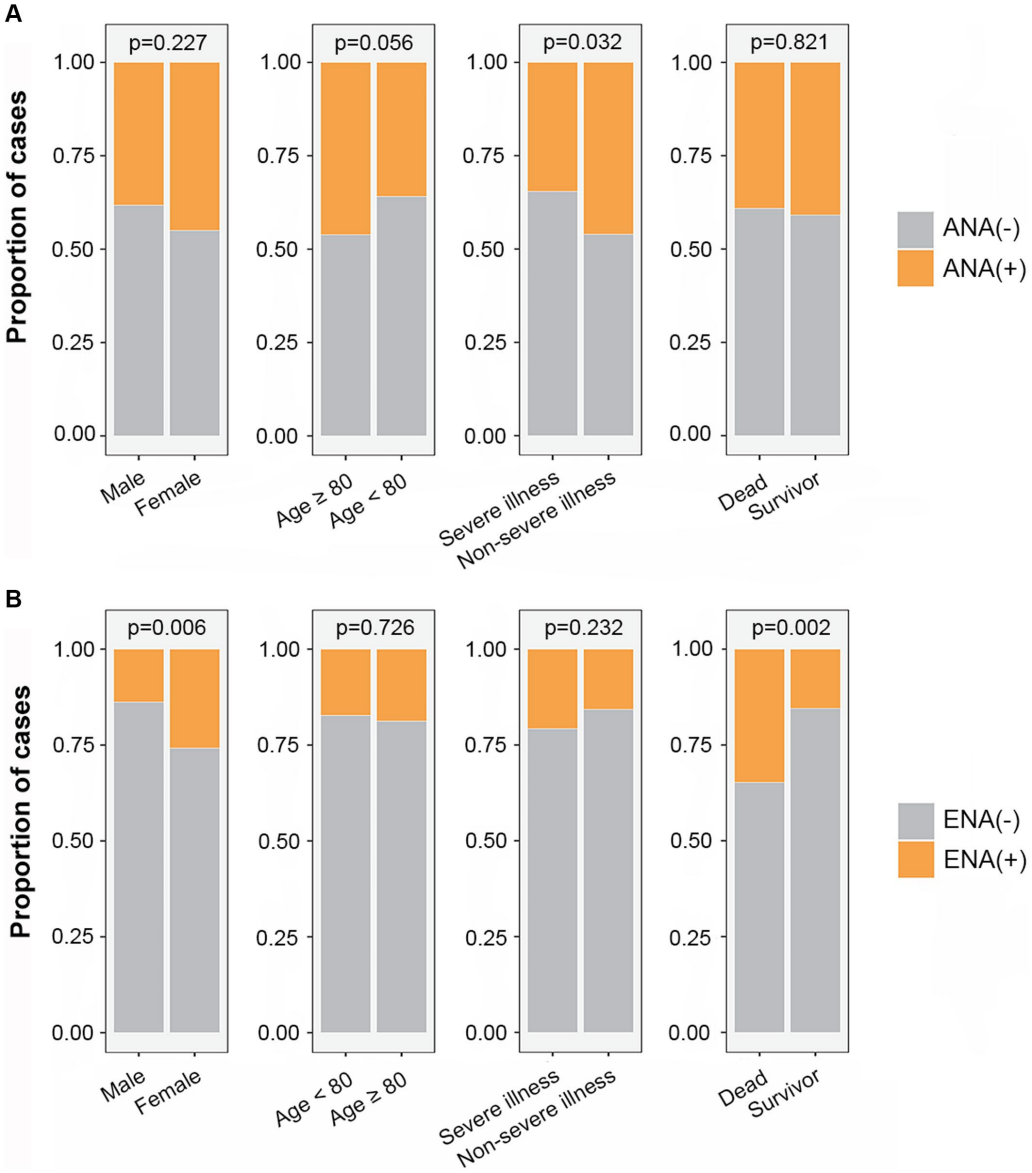
The proportion of ANA positivity **(A)** and ENA positivity **(B)** in different subgroups. ANA, antinuclear antibody; ENA, anti-extractable nuclear antigen antibody.

ANA and ENA positivity were compared between different groups divided by sex, age, disease severity, and outcomes of COVID-19 patients. The result showed that the proportion of ANA positivity was higher in non-severe illness group (Figure 1A) and the proportion of ENA positivity was higher in females than in males, and was higher in dead COVID-19 patients than in discharged patients (Figure 1B).

### 3.3 Univariate and multivariate logistic regression analyses of the related factors of disease severity of COVID-19

The risk or protective factors for the disease severity of COVID-19 were identified by univariate and multivariate logistic regression analyses. The univariate logistic regression analysis showed that age (OR = 1.029; 95% CI, 1.013–1.045; *p* < 0.001), WBC (OR = 1.108; 95% CI, 1.050–1.169; *p* < 0.001), aspartate aminotransferase (AST) (OR = 1.013; 95% CI, 1.001–1.026; *p* = 0.037), C reactive proteins (CRP) (OR = 1.014; 95% CI, 1.008–1.020; *p* < 0.001), and ANA positivity (OR = 0.619; 95% CI, 0.399–0.961; *p* = 0.033) were associated with the disease severity of COVID-19. The multivariate logistic regression analysis showed that age (OR = 1.026; 95% CI, 1.008–1.043; *p* = 0.004), WBC (OR = 1.087; 95% CI, 1.028–1.151; *p* = 0.004), and CRP (OR = 1.010; 95% CI, 1.004–1.017; *p* = 0.001) were independent risk factors for disease severity of COVID-19, while the ANA positivity (OR = 0.560; 95% CI, 0.348–0.902; *p* = 0.017) was an independent protective factor (Table 2).

**Table 2:** Univariate and multivariate logistic regression analyses of the related factors of the disease severity of COVID-19.

Variables	Univariate analysis		Multivariate analysis	
	OR (95% CI)	*p*-value	OR (95% CI)	*p-*value
Age (years)	1.029 (1.013,1.045)	**0.000**	1.026 (1.008,1.043)	**0.004**
Sex				
Female	Reference			
Male	1.413 (0.901,2.216)	0.132		
Hypertension	1.192 (0.777,1.829)	0.422		
Diabetes	1.316 (0.826,2.097)	0.247		
Malignancy	0.561 (0.302,1.042)	0.067		
WBC (1 × 10^9^/L)	1.108 (1.050,1.169)	**0.000**	1.087 (1.028,1.151)	**0.004**
ALT (U/L)	1.000 (0.993,1.007)	0.981		
AST (U/L)	1.013 (1.001,1.026)	**0.037**	1.011 (0.998,1.024)	0.094
CREA (μmol/L)	1.002 (1.000,1.003)	0.097		
CRP (mg/L)	1.014 (1.008,1.020)	**0.000**	1.010 (1.004,1.017)	**0.001**
ANA				
Negative	Reference			
Positive	0.619 (0.399,0.961)	**0.033**	0.560 (0.348,0.902)	**0.017**
ENA				
Negative	Reference			
Positive	1.403 (0.804,2.448)	0.233		

ALT, alanine aminotransferase; ANA, antinuclear antibody; AST, aspartate aminotransferase; CREA, creatinine; CRP, c-reactive protein; ENA, anti-extractable nuclear antigen antibody; WBC, white blood cell.

### 3.4 Univariate and multivariate Cox regression analyses of the related factors of outcome of COVID-19 patients

The Cox regression analysis was conducted to confirm the impact of ANA and ENA positivity on the COVID-19 patients’ prognosis. The univariate analysis showed that WBC (HR = 1.033; 95% CI, 1.021–1.044; *p* < 0.001), AST (HR = 1.021; 95% CI, 1.012–1.031; *p* < 0.001), CREA (HR = 1.001; 95% CI, 1.000–1.003; *p* = 0.018), CRP (HR = 1.013; 95% CI, 1.007–1.019; *p* < 0.001), and ENA positivity (HR = 2.575; 95% CI, 1.399–4.739; *p* = 0.002) were related to the outcomes of COVID-19 patients (Table 3). The multivariate analysis showed that WBC (HR = 1.033; 95% CI; 1.021–1.045; *p* < 0.001), AST (HR = 1.027; 95% CI, 1.017–1.037; *p* < 0.001), CREA (HR = 1.001; 95% CI, 1.000–1.003; *p* = 0.027), CRP (HR = 1.011; 95% CI, 1.005–1.018; *p* = 0.001), and ENA positivity (HR = 2.477; 95% CI, 1.321–4.646; *p* = 0.005) were independent risk factors for the COVID-19 patients’ prognosis (Table 3).

**Table 3:** Univariate and multivariate Cox regression analyses of the related factors to the death of COVID-19 patients.

Variables	Univariate analysis		Multivariate analysis	
	HR (95% CI)	*p*-value	HR (95% CI)	*p-*value
Age (years)	1.014 (0.991,1.038)	0.233		
Sex				
Female	Reference			
Male	0.856 (0.470,1.558)	0.610		
Hypertension	1.031 (0.578,1.839)	0.918		
Diabetes	1.175 (0.645,2.142)	0.598		
Malignancy	0.892 (0.378,2.107)	0.795		
WBC (1 × 10^9^/L)	1.033 (1.021,1.044)	**0.000**	1.033 (1.021,1.045)	**0.000**
ALT (U/L)	1.007 (1.000,1.015)	0.062		
AST (U/L)	1.021 (1.012,1.031)	**0.000**	1.027 (1.017,1.037)	**0.000**
CREA (μmol/L)	1.001 (1.000,1.003)	**0.018**	1.001 (1.000,1.003)	**0.027**
CRP (mg/L)	1.013 (1.007,1.019)	**0.000**	1.011 (1.005,1.018)	**0.000**
ANA				
Negative	Reference			
Positive	1.034 (0.571,1.872)	0.913		
ENA				
Negative	Reference			
Positive	2.575 (1.399,4.739)	**0.002**	2.477 (1.321,4.646)	**0.005**

ALT, alanine aminotransferase; ANA, antinuclear antibody; AST, aspartate aminotransferase; CREA, creatinine; CRP, c-reactive protein; ENA, anti-extractable nuclear antigen antibody; WBC, white blood cell.

### 3.5 A reduced survival rate in COVID-19 patients with ENA positivity

**Figure 2:**
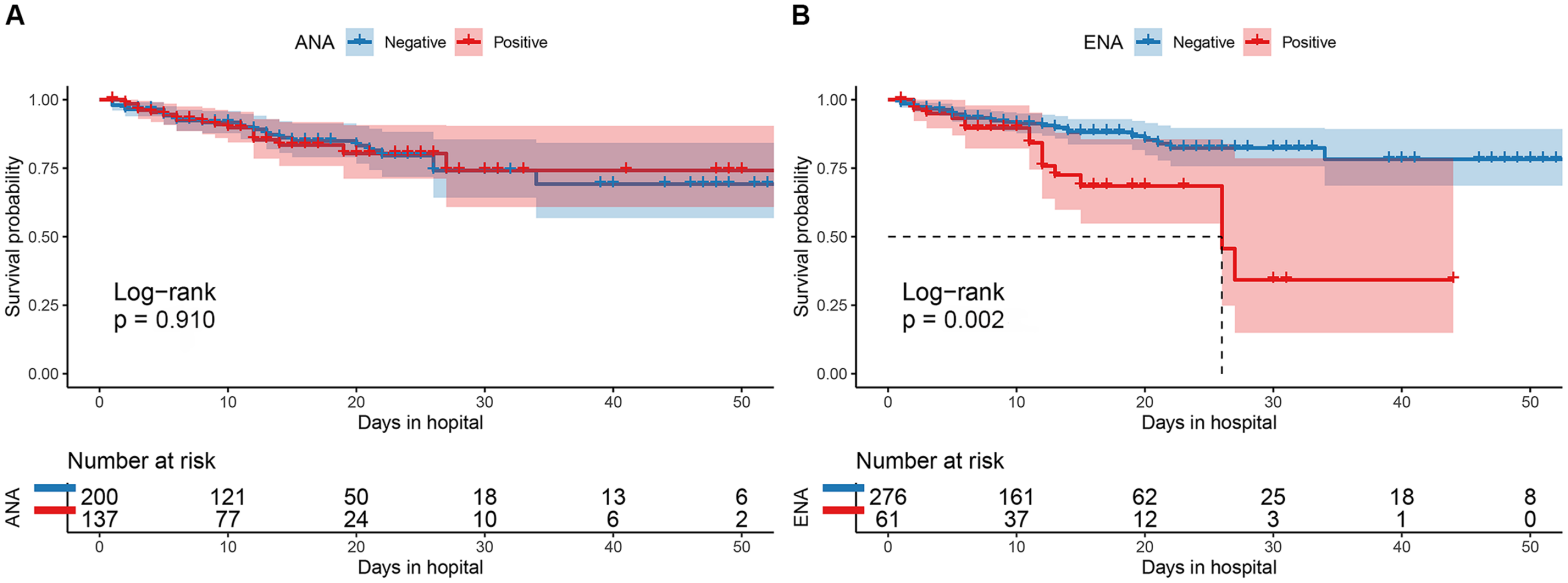
Comparison of the cumulative survival rate between patients with and without ANA positivity **(A)** and between those with and without ENA positivity **(B)**. ANA, antinuclear antibody; ENA, anti-extractable nuclear antigen antibody.

**Figure 3:**
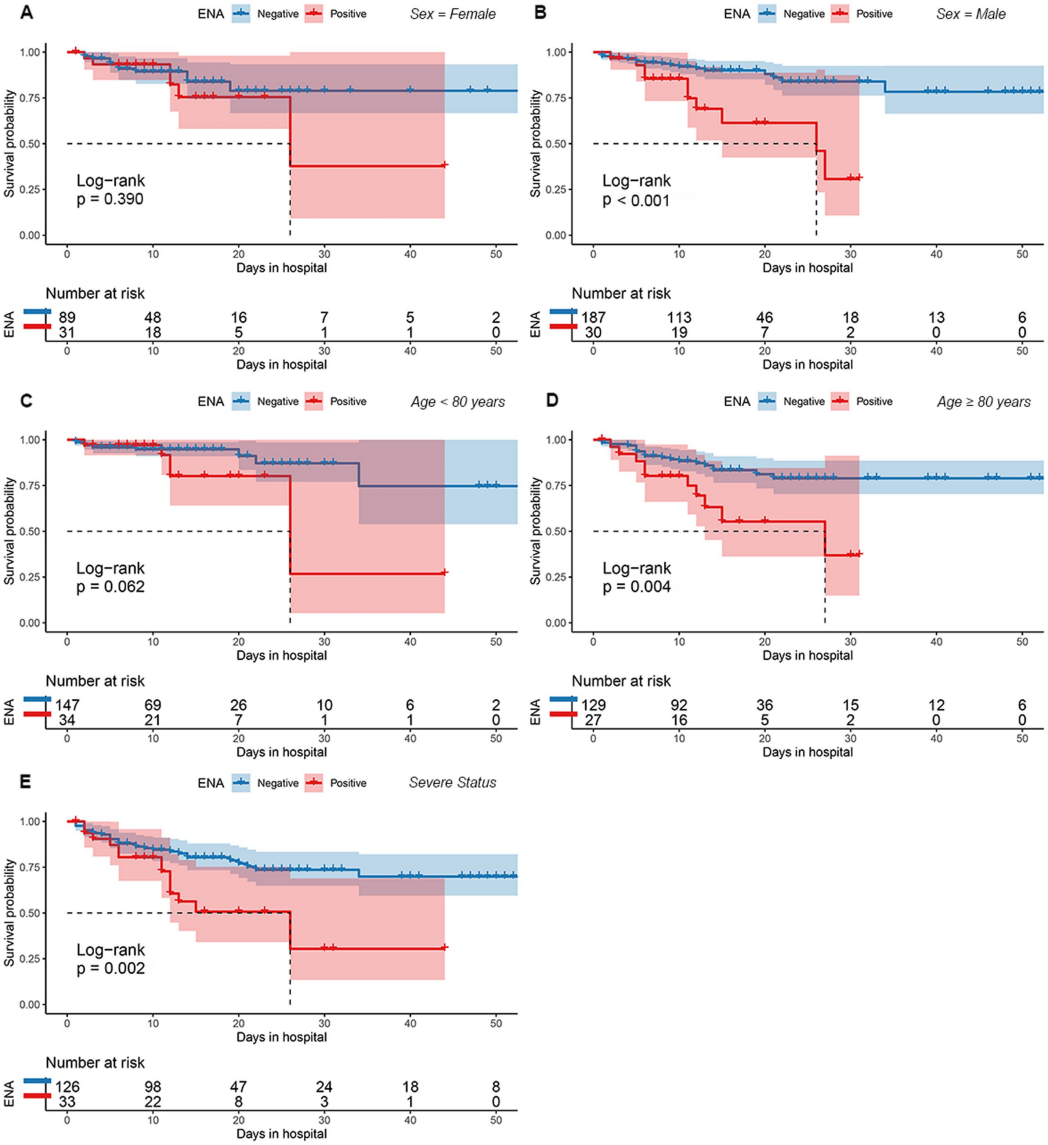
The cumulative survival rate for between patients with and without ENA positivity for various subgroups based on sex **(A,B)**, age **(C,D)**, and degree of disease severity **(E)**. ENA, anti-extractable nuclear antigen antibody.

There were 30 males and 31 females in ENA-positive COVID-19 patients, compared to 187 males and 89 females in ENA-negative patients. Age ≥ 80 years old were present in 44% of the ENA-positive COVID-19 patients, compared to 47% of the ENA-negative COVID-19 patients. There were 54% of the patients had severe illness in the ENA-positive group, compared to 46% in the ENA-negative group. The median days of hospitalization of those 337 COVID-19 patients were 11 (6–16) days. There were 46 (13.6%) deaths during the hospitalization, including 30 ENA-negative COVID-19 patients and 16 ENA-positive COVID-19 patients. The Kaplan–Meier survival curves showed that ENA-positive COVID-19 patients had a lower cumulative survival rate compared with ENA-negative patients (*p* = 0.002; Figure 2B). Since studies have concluded that the outcome of COVID-19 was affected by various of factors, such as age, disease severity, and complicating with chronic diseases, we further analyzed the prognostic role of the ENA positivity in subgroups divided by age (>80 years), sex, and disease severity. The result showed that ENA positivity was still correlated with a lower cumulative survival rate in males, age ≥ 80 years old, and severe COVID-19 patients (Figures 3B,D,E), whereas the impact of ENA positivity on prognosis was mild in groups of female and age < 80 years old (Figures 3A,C).

## 4 Discussion

In our single-center study, we enrolled 337 hospitalized COVID-19 patients, of whom 159 (47%) patients had severe or critical COVID-19 and 178 (53%) patients had mild or moderate COVID-19. We found that 47% of COVID-19 patients expressed ANA or ENA in the serum, and 11% of COVID-19 patients expressed both ANA and ENA. The proportion of ANA positivity was higher in non-severe illness group. The multivariate logistic regression analysis showed that ANA positivity was an independent protective factor for the disease severity of COVID-19. Compared with ENA-negative patients, ENA-positive patients died more during hospitalization, further multivariate Cox regression analysis showed that ENA positivity was an independent risk factor for the outcome of COVID-19 patients with ENA-positive COVID-19 patients have a lower cumulative survival rate.

Genetical, environmental, and hormonal factors are suggested to be the three critical causes for the occurrence of autoimmune diseases (Shoenfeld et al., 2008a,b,c). Viral infection has been identified as a principal environmental factor inducing autoimmunity (Getts et al., 2013), and the mechanisms may involve the molecular resemblance between virus and self-antigens, epitope spreading, and bystander T cell activation (Oldstone, 1998; Fujinami et al., 2006; Smatti et al., 2019; Galeotti and Bayry, 2020). COVID-19 is characterized as a hyperinflammation syndrome triggered by a cytokine storm. The excessive innate immune response and the hyperactivation of adaptive immune response which results in acute extrafollicular expansion of autoimmune-like B cells and the intensive secretion of autoantibodies (Woodruff et al., 2020; Wang et al., 2021a). ANA positivity was detected in 41% (137/337) of COVID-19 patients in our study. The proportion of ANA positivity was higher in patients classified into non-severe illness group than in those classified into severe illness group. COVID-19 patients with different degrees of disease severity had distinct ANA expressions, suggesting that ANA may shed a new light on the immunopathogenesis of COVID-19. Therefore, further studies focus on the mechanisms of ANA in the pathogenesis of COVID-19 may help to improve the treatment of COVID-19 patients. A number of researches have revealed that COVID-19 patients had a positive ENA, and some have found that ENA positivity was correlated with severe or critical COVID-19 and high mortality in COVID-19 patients (Zhou Y. et al., 2020; Peker et al., 2021; Son et al., 2023; Vahabi et al., 2023). Our present study showed that ENA positivity was associated with the outcome of COVID-19 patients and that COVID-19 patients who died during hospitalization had a higher proportion of ENA positivity than convalescent patients. Besides, we found that IL-6 level was higher in ENA-positive patients than in ENA-negative patients. Previous studies have demonstrated that infection of SARS-CoV-2 could induce the secretion of IL-6. IL-6 and other inflammatory cytokines may cause autoimmunity through systemic inflammation (Atabati et al., 2020; Picchianti et al., 2020; Xu et al., 2020). Indeed, IL-6 was also found associated with the positivity of autoantibodies in other studies (Pascolini et al., 2021; Son et al., 2023).

Previous studies suggested that the proportion of ANA positivity was higher in patients with severe or critical COVID-19 than in patients with mild or moderate COVID-19 and the prognosis of ANA-positive patients was worse (Peker et al., 2021; Brianza-Padilla et al., 2022; Nasarallah et al., 2022). However, in the present study, the multivariate logistic regression showed that ANA positivity was an independent protective factor for the disease severity of COVID-19, while age, WBC, and CRP were independent risk factors for the disease severity of COVID-19. Despite our finding is provocative, researchers demonstrated that ANAs were also present in other acute and chronic infectious and usually not associated with an autoimmune disease (Litwin and Binder, 2016; Woodruff et al., 2022). In addition, researchers found that some autoantibodies with ANA reactivity also had some affinity to a screened viral antigen in COVID-19 patients (Woodruff et al., 2022). This suggests that these autoantibodies may also contribute to the neutralization of virus to some extent. Besides, Peker et al. found that the ratio of ANA-positive patients was higher in clinic than in ICU (Peker et al., 2021). Moreover, Peker et al. and Vahabi et al. found that the titer of ANA was higher in non-ICU COVID-19 patients than in COVID-19 patients admitted to the ICU (Peker et al., 2021; Vahabi et al., 2023). Therefore, the correlation between ANA positivity and COVID-19 severity needs to be further explored in future studies. The present study did not find an association between ANA positivity and the outcomes of COVID-19 patients.

The Cox regression analysis showed that ENA positivity was an independent risk factor for the COVID-19 patients’ prognosis. Kaplan–Meier survival curves showed that ENA-positive COVID-19 patients had a lower cumulative survival rate during hospitalization. Several risk factors, including male, old age, and pre-existing comorbidities, have been identified to cause high mortality in COVID-19 patients (Fauci et al., 2020). Therefore, we further analyzed the effect of ENA positivity on the prognosis of patients in the subgroups based on sex, age, and disease severity. The result showed that ENA positivity was still correlated lower cumulative survival rate in males, patients ≥80 years old, or patients with severe COVID-19. This further consolidates the association between the ENA positivity with the lower cumulative survival rate in COVID-19 patients. In COVID-19 patients autoantibodies participate tissue damaging and promote disease progression through mechanisms including complement activation, cytokine neutralization, disrupting cytokine signaling, damaging cell-to-cell adhesion, antagonizing receptor activity, and triggering neutrophil hyper-reactivity (Bastard et al., 2020; Gruber et al., 2020; Zuo et al., 2020; Wang et al., 2021b). However, reports about the role of ENA positivity in COVID-19 remained inconsistent among published studies (Zhou Y. et al., 2020; Peker et al., 2021; Son et al., 2023; Vahabi et al., 2023). Therefore, further studies are needed to explore the exact pathological mechanism of ENA in COVID-19.

The present study several limitations. First, the numbers of enrolled COVID-19 patients in our study were relatively small. Thus, multi-center studies with a larger sample size were required to validate our findings. Second, we only divided patients into mild or moderate and severe or critical COVID-19 groups. To clarify the role of autoantibodies in the disease progression of COVID-19, future researches should make detailed groupings, including mild, moderate, severe, and critical COVID-19.

In summary, we concluded that a spectrum of autoantibodies was expressed in COVID-19 patients and that ANA and ENA positivity were associated with disease severity and the prognosis of COVID-19 patients, respectively. Therefore, autoantibodies may play a role in assessing the disease severity and prognosis of COVID-19 patients and may help to understand the pathogenesis of COVID-19 from a new perspective.

## Data availability statement

The original contributions presented in the study are included in the article/Supplementary material, further inquiries can be directed to the corresponding authors.

## Ethics statement

The studies involving humans were approved by Ethics Committee of Nanjing Drum Tower Hospital. The studies were conducted in accordance with the local legislation and institutional requirements. The participants provided their written informed consent to participate in this study.

## Author contributions

WeiZ: Conceptualization, Formal analysis, Investigation, Methodology, Visualization, Writing – original draft, Writing – review & editing. YT: Conceptualization, Formal analysis, Methodology, Validation, Writing – original draft, Writing – review & editing. YZ: Formal analysis, Methodology, Validation, Writing – review & editing. QZ: Investigation, Methodology, Data curation, Writing – review & editing. FH: Methodology, Validation, Data curation, Writing – review & editing. WenZ: Conceptualization, Data curation, Methodology, Supervision, Validation, Writing – review & editing. JW: Conceptualization, Data curation, Formal analysis, Methodology, Supervision, Writing – review & editing. MN: Conceptualization, Data curation, Funding acquisition, Investigation, Project administration, Resources, Supervision, Writing – review & editing.

## References

[ref1] Atabati H. Esmaeili S. A. Saburi E. Akhlaghi M. Raoofi A. Rezaei N.. (2020). Probiotics with ameliorating effects on the severity of skin inflammation in psoriasis: evidence from experimental and clinical studies. J. Cell. Physiol. 235, 8925–8937. doi: 10.1002/jcp.29737, PMID: 32346892

[ref2] Bastard P. Rosen L. B. Zhang Q. Michailidis E. Hoffmann H. H. Zhang Y. (2020). Autoantibodies against type i ifns in patients with life-threatening COVID-19. Science 370. doi: 10.1126/science.abd4585, PMID: 32972996 PMC7857397

[ref3] Brianza-Padilla M. Juárez-Vicuña Y. Springall R. González-Flores J. Patlán M. Amezcua-Guerra L. M. (2022). Antinuclear antibodies detected by enzyme-linked immunosorbent assay (ELISA) in severe COVID-19: clinical and laboratory associations. Eur. Rev. Med. Pharmacol. Sci. 26, 5307–5310. doi: 10.26355/eurrev_202207_29322, PMID: 35916831

[ref4] Chang S. E. Feng A. Meng W. Apostolidis S. A. Mack E. Artandi M. (2021). New-onset igg autoantibodies in hospitalized patients with COVID-19. Nat. Commun. 12:5417. doi: 10.1038/s41467-021-25509-3, PMID: 34521836 PMC8440763

[ref915] Damoiseaux J. von Mühlen C. A. Garcia-De La Torre I. Carballo O. G. de Melo Cruvinel W. Francescantonio P. L.. (2016). International consensus on ANA patterns (ICAP): the bumpy road towards a consensus on reporting ANA results. Auto Immun. Highlights. 7:1. doi: 10.1007/s13317-016-0075-0 26831867 PMC4733811

[ref5] Esmaeili A. Rabe S. Mahmoudi M. Rastin M. (2017). Frequencies of hla-a, b and drb1 alleles in a large normal population living in the city of Mashhad, northeastern Iran. Iran. J. Basic Med. Sci. 20, 940–943. doi: 10.22038/IJBMS.2017.9117, PMID: 29085586 PMC5651480

[ref6] Fauci A. S. Lane H. C. Redfield R. R. (2020). COVID-19 – navigating the uncharted. N. Engl. J. Med. 382, 1268–1269. doi: 10.1056/NEJMe2002387, PMID: 32109011 PMC7121221

[ref7] Fujinami R. S. von Herrath M. G. Christen U. Whitton J. L. (2006). Molecular mimicry, bystander activation, or viral persistence: infections and autoimmune disease. Clin. Microbiol. Rev. 19, 80–94. doi: 10.1128/CMR.19.1.80-94.2006, PMID: 16418524 PMC1360274

[ref8] Galeotti C. Bayry J. (2020). Autoimmune and inflammatory diseases following COVID-19. Nat. Rev. Rheumatol. 16, 413–414. doi: 10.1038/s41584-020-0448-7, PMID: 32499548 PMC7271827

[ref9] Getts D. R. Chastain E. M. Terry R. L. Miller S. D. (2013). Virus infection, antiviral immunity, and autoimmunity. Immunol. Rev. 255, 197–209. doi: 10.1111/imr.12091, PMID: 23947356 PMC3971377

[ref10] Gruber C. N. Patel R. S. Trachtman R. Lepow L. Amanat F. Krammer F.. (2020). Mapping systemic inflammation and antibody responses in multisystem inflammatory syndrome in children (mis-c). Cells 183, 982–995.e14. doi: 10.1016/j.cell.2020.09.034, PMID: 32991843 PMC7489877

[ref11] Gugliesi F. Pasquero S. Griffante G. Scutera S. Albano C. Pacheco S.. (2021). Human cytomegalovirus and autoimmune diseases: where are we? Viruses 13:260. doi: 10.3390/v13020260, PMID: 33567734 PMC7914970

[ref12] Hu B. Huang S. Yin L. (2021). The cytokine storm and COVID-19. J. Med. Virol. 93, 250–256. doi: 10.1002/jmv.26232, PMID: 32592501 PMC7361342

[ref13] Jakhmola S. Upadhyay A. Jain K. Mishra A. Jha H. C. (2021). Herpesviruses and the hidden links to multiple sclerosis neuropathology. J. Neuroimmunol. 358:577636. doi: 10.1016/j.jneuroim.2021.577636, PMID: 34174587

[ref14] Kronstein-Wiedemann R. Stadtmüller M. Traikov S. Georgi M. Teichert M. Yosef H.. (2022). SARS-cov-2 infects red blood cell progenitors and dysregulates hemoglobin and iron metabolism. Stem Cell Rev. Rep. 18, 1809–1821. doi: 10.1007/s12015-021-10322-8, PMID: 35181867 PMC8856880

[ref15] Litwin C. M. Binder S. R. (2016). Ana testing in the presence of acute and chronic infections. J. Immunoassay Immunochem. 37, 439–452. doi: 10.1080/15321819.2016.1174136, PMID: 27050929

[ref16] Luo X. H. Zhu Y. Mao J. Du R. C. (2021). T cell immunobiology and cytokine storm of COVID-19. Scand. J. Immunol. 93:e12989. doi: 10.1111/sji.12989, PMID: 33113222 PMC7645942

[ref17] Nasarallah G. K. Fakhroo A. D. Khan T. Cyprian F. S. Al A. F. Ata M.. (2022). Detection of antinuclear antibodies targeting intracellular signal transduction, metabolism, apoptotic processes and cell death in critical COVID-19 patients. Mediterr. J. Hematol. Infect. Dis. 14:e2022076. doi: 10.4084/MJHID.2022.076, PMID: 36425144 PMC9652015

[ref18] Oldstone M. B. (1998). Molecular mimicry and immune-mediated diseases. FASEB J. 12, 1255–1265. doi: 10.1096/fasebj.12.13.1255, PMID: 9761770 PMC7164021

[ref19] Pascolini S. Vannini A. Deleonardi G. Ciordinik M. Sensoli A. Carletti I.. (2021). COVID-19 and immunological dysregulation: can autoantibodies be useful? Clin. Transl. Sci. 14, 502–508. doi: 10.1111/cts.12908, PMID: 32989903 PMC7536986

[ref20] Peker B. O. Şener A. G. Kaptan A. F. (2021). Antinuclear antibodies (ANAs) detected by indirect immunofluorescence (IIF) method in acute COVID-19 infection; future roadmap for laboratory diagnosis. J. Immunol. Methods 499:113174. doi: 10.1016/j.jim.2021.113174, PMID: 34737165 PMC8556075

[ref21] Picchianti D. A. Rosado M. M. Pioli C. Sesti G. Laganà B. (2020). Cytokine release syndrome in COVID-19 patients, a new scenario for an old concern: the fragile balance between infections and autoimmunity. Int. J. Mol. Sci. 21:3330. doi: 10.3390/ijms21093330 32397174 PMC7247555

[ref22] Quaglia M. Merlotti G. De Andrea M. Borgogna C. Cantaluppi V. (2021). Viral infections and systemic lupus erythematosus: new players in an old story. Viruses 13. doi: 10.3390/v13020277, PMID: 33670195 PMC7916951

[ref23] Scappaticcio L. Pitoia F. Esposito K. Piccardo A. Trimboli P. (2021). Impact of COVID-19 on the thyroid gland: an update. Rev. Endocr. Metab. Disord. 22, 803–815. doi: 10.1007/s11154-020-09615-z, PMID: 33241508 PMC7688298

[ref24] Schaller T. Hirschbühl K. Burkhardt K. Braun G. Trepel M. Märkl B.. (2020). Postmortem examination of patients with COVID-19. JAMA 323, 2518–2520. doi: 10.1001/jama.2020.8907, PMID: 32437497 PMC7243161

[ref25] Shoenfeld Y. Blank M. Abu-Shakra M. Amital H. Barzilai O. Berkun Y.. (2008a). The mosaic of autoimmunity: prediction, autoantibodies, and therapy in autoimmune diseases--2008. Isr. Med. Assoc. J. 10, 13–19. PMID: 18300564

[ref26] Shoenfeld Y. Gilburd B. Abu-Shakra M. Amital H. Barzilai O. Berkun Y.. (2008b). The mosaic of autoimmunity: genetic factors involved in autoimmune diseases--2008. Isr. Med. Assoc. J. 10, 3–7. PMID: 18300562

[ref27] Shoenfeld Y. Zandman-Goddard G. Stojanovich L. Cutolo M. Amital H. Levy Y.. (2008c). The mosaic of autoimmunity: hormonal and environmental factors involved in autoimmune diseases--2008. Isr. Med. Assoc. J. 10, 8–12. PMID: 18300563

[ref28] Smatti M. K. Cyprian F. S. Nasrallah G. K. Al T. A. Almishal R. O. Yassine H. M. (2019). Viruses and autoimmunity: a review on the potential interaction and molecular mechanisms. Viruses 11:762. doi: 10.3390/v11080762, PMID: 31430946 PMC6723519

[ref29] Son K. Jamil R. Chowdhury A. Mukherjee M. Venegas C. Miyasaki K.. (2023). Circulating anti-nuclear autoantibodies in COVID-19 survivors predict long COVID symptoms. Eur. Respir. J. 61. doi: 10.1183/13993003.00970-2022, PMID: 36137590 PMC9515477

[ref30] Stjepanovic M. I. Stojanovic M. R. Stankovic S. Cvejic J. Dimic-Janjic S. Popevic S.. (2022). Autoimmune and immunoserological markers of COVID-19 pneumonia: can they help in the assessment of disease severity. Front. Med. 9:934270. doi: 10.3389/fmed.2022.934270, PMID: 36106319 PMC9464912

[ref31] Ulndreaj A. Wang M. Misaghian S. Paone L. Sigal G. B. Stengelin M.. (2022). Patients with severe COVID-19 do not have elevated autoantibodies against common diagnostic autoantigens. Clin. Chem. Lab. Med. 60, 1116–1123. doi: 10.1515/cclm-2022-0239, PMID: 35475723 PMC9128368

[ref32] Vahabi M. Mirsharif E. S. Ghazanfari T. (2023). Is COVID-19 severity unrelated to antinuclear antibodies? Transpl. Immunol. 78:101791. doi: 10.1016/j.trim.2023.101791, PMID: 36682573 PMC9851722

[ref33] Viruses C. S. G. O. (2020). The species severe acute respiratory syndrome-related coronavirus: classifying 2019-ncov and naming it SARS-cov-2. Nat. Microbiol. 5, 536–544. doi: 10.1038/s41564-020-0695-z, PMID: 32123347 PMC7095448

[ref34] Wang E. Y. Mao T. Klein J. Dai Y. Huck J. D. Jaycox J. R.. (2021a). Diverse functional autoantibodies in patients with COVID-19. Nature 595, 283–288. doi: 10.1038/s41586-021-03631-y, PMID: 34010947 PMC13130511

[ref35] Wang E. Y. Mao T. Klein J. Dai Y. Huck J. D. Liu F.. (2021b). Diverse functional autoantibodies in patients with COVID-19. medRxiv. doi: 10.1101/2020.12.10.20247205, PMID: 34010947 PMC13130511

[ref36] Wong M. K. Brooks D. J. Ikejezie J. Gacic-Dobo M. Dumolard L. Nedelec Y.. (2023). COVID-19 mortality and progress toward vaccinating older adults – world health organization, worldwide, 2020–2022. MMWR Morb. Mortal. Wkly Rep. 72, 113–118. doi: 10.15585/mmwr.mm7205a1, PMID: 36730046 PMC9927068

[ref37] Woodruff M. C. Ramonell R. P. Haddad N. S. Anam F. A. Rudolph M. E. Walker T. A.. (2022). Dysregulated naive b cells and de novo autoreactivity in severe COVID-19. Nature 611, 139–147. doi: 10.1038/s41586-022-05273-0, PMID: 36044993 PMC9630115

[ref38] Woodruff M. C. Ramonell R. P. Nguyen D. C. Cashman K. S. Saini A. S. Haddad N. S.. (2020). Extrafollicular B cell responses correlate with neutralizing antibodies and morbidity in COVID-19. Nat. Immunol. 21, 1506–1516. doi: 10.1038/s41590-020-00814-z, PMID: 33028979 PMC7739702

[ref39] Xu Z. Shi L. Wang Y. Zhang J. Huang L. Zhang C.. (2020). Pathological findings of COVID-19 associated with acute respiratory distress syndrome. Lancet Respir. Med. 8, 420–422. doi: 10.1016/S2213-2600(20)30076-X, PMID: 32085846 PMC7164771

[ref40] Zhou Y. Han T. Chen J. Hou C. Hua L. He S.. (2020). Clinical and autoimmune characteristics of severe and critical cases of COVID-19. Clin. Transl. Sci. 13, 1077–1086. doi: 10.1111/cts.12805, PMID: 32315487 PMC7264560

[ref41] Zhou P. Yang X. L. Wang X. G. Hu B. Zhang L. Zhang W.. (2020). A pneumonia outbreak associated with a new coronavirus of probable bat origin. Nature 579, 270–273. doi: 10.1038/s41586-020-2012-7, PMID: 32015507 PMC7095418

[ref42] Zuo Y. Estes S. K. Ali R. A. Gandhi A. A. Yalavarthi S. Shi H.. (2020). Prothrombotic autoantibodies in serum from patients hospitalized with COVID-19. Sci. Transl. Med. 12. doi: 10.1126/scitranslmed.abd3876, PMID: 33139519 PMC7724273

