# Supplementary figures and images for: Effect of serum autoantibodies on the COVID-19 patient’s prognosis
Source: Front Microbiol. 2023 Nov 30;14:1259960. doi: 10.3389/fmicb.2023.1259960 (PMC10721969; doi:10.3389/fmicb.2023.1259960)

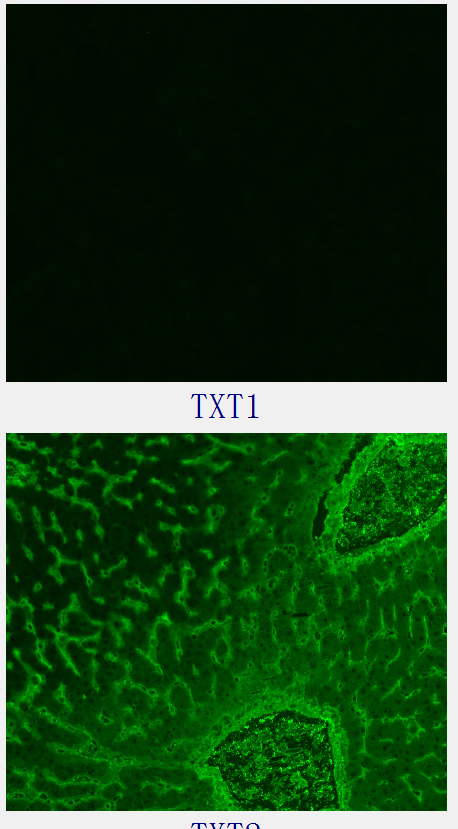

Supplement: Supplementary file 1 [file Image_1.PNG]

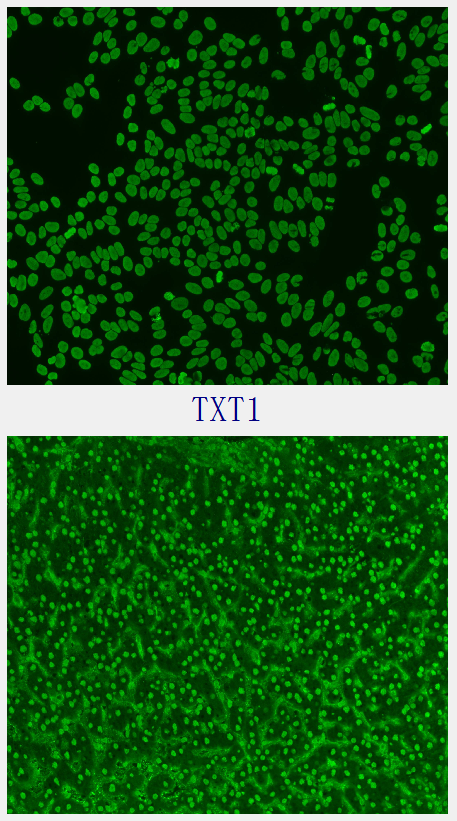

Supplement: Supplementary file 2 [file Image_2.PNG]

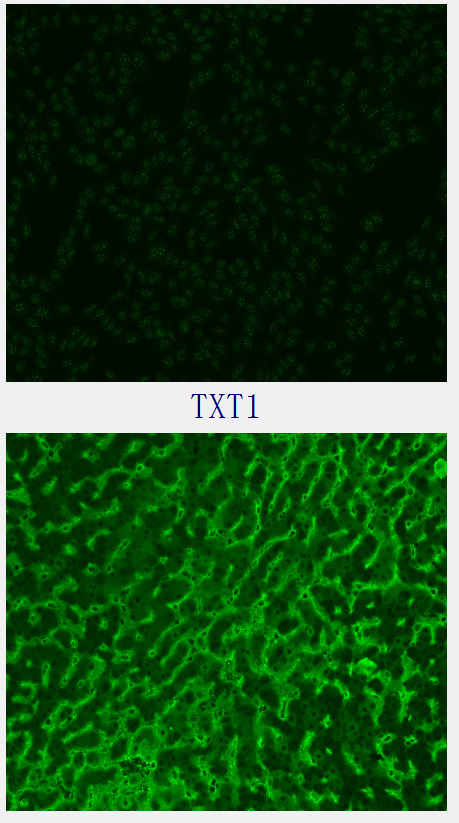

Supplement: Supplementary file 3 [file Image_3.PNG]

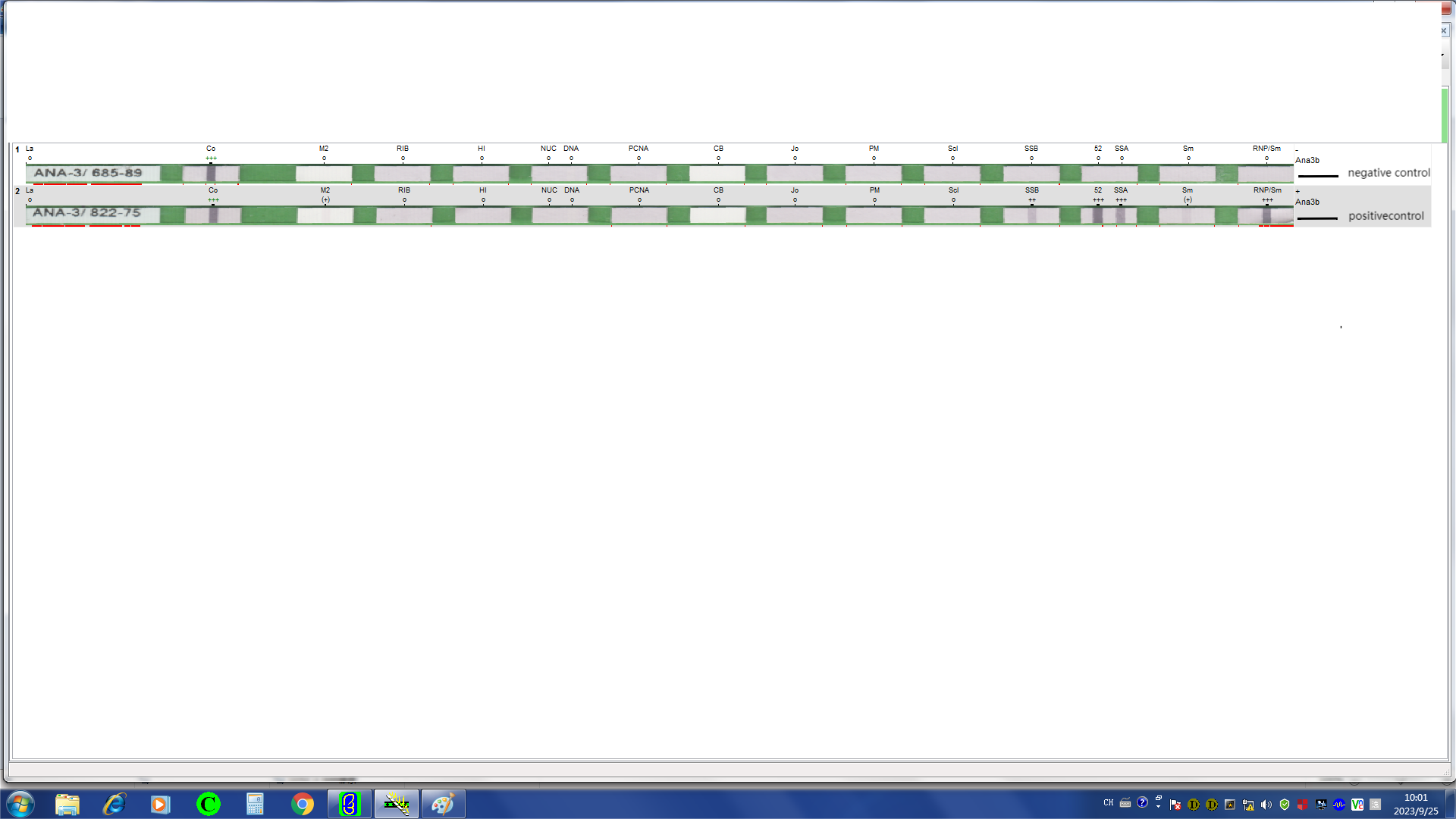

Supplement: Supplementary file 4 [file Image_4.PNG]
