# Supplementary material for: Effect of serum autoantibodies on the COVID-19 patient’s prognosis
Source: Front Microbiol. 2023 Nov 30;14:1259960. doi: 10.3389/fmicb.2023.1259960 (PMC10721969; doi:10.3389/fmicb.2023.1259960)
Supplement: Supplementary file 5 [file Table_1.DOCX]

**Table S1. Univariate and multivariate logistic regression analyses of the related factors of the disease severity of COVID-19 without hypertension**

| **Variables** | **Univariate analysis** | | **Multivariate analysis** | |
| --- | --- | --- | --- | --- |
|  | OR (95% CI) | *P*-value | OR (95% CI) | *P-*value |
| Age (years) | 1.039(1.017,1.062) | **0.001** | 1.039(1.013,1.065) | **0.003** |
| Sex |  |  |  |  |
| Female | reference |  |  |  |
| Male | 1.524(0.804,2.890) | 0.197 |  |  |
| Diabetes | 1.848(0.872,3.917) | 0.109 |  |  |
| Malignancy | 0.706(0.327,1.523) | 0.375 |  |  |
| WBC (1×10^9^/L) | 1.099(1.016,1.188) | **0.018** | 1.100(1.004,1.207) | **0.041** |
| ALT (U/L) | 0.998(0.989,1.007) | 0.726 |  |  |
| AST (U/L) | 1.012(0.997,1.028) | 0.121 |  |  |
| CREA (μmol/L) | 1.014(1.003,1.025) | **0.012** | 1.010(0.999,1.021) | 0.080 |
| CRP (mg/L) | 1.021(1.012,1.030) | **0.000** | 1.014(1.005,1.024) | **0.004** |
| ANA |  |  |  |  |
| negative | Reference |  |  |  |
| positive | 0.509(0.276,0.941) | **0.031** | 0.379(0.184,0.781) | **0.009** |
| ENA |  |  |  |  |
| negative | Reference |  |  |  |
| positive | 1.848(0.872,3.917) | 0.109 |  |  |

**Table S2. The AC patterns for ANA in COVID-19 patients.**

| AC pattern | All | Severe illness | Non-severe illness |
| --- | --- | --- | --- |
| AC-4, 5 | 42 | 18 | 24 |
| AC-4, 5 and AC-19, 20 | 23 | 8 | 15 |
| AC-1 | 15 | 7 | 8 |
| AC-8, 9, 10 | 13 | 6 | 7 |
| AC-19, 20 | 13 | 3 | 10 |
| AC-3 | 4 |  | 4 |
| AC-15, 16, 17 | 4 | 1 | 3 |
| AC-3 and AC-19,20 | 3 | 3 |  |
| AC-1 and AC-19, 20 | 3 |  | 3 |
| AC-25 | 3 | 1 | 2 |
| AC-15, 16, 17 and AC-4, 5 | 3 | 3 |  |
| AC-4, 5 and AC-8, 9, 10 | 2 | 1 | 1 |
| AC-1 and AC-23 | 2 |  | 2 |
| AC-18 | 2 | 2 |  |
| AC-23 | 2 | 1 | 1 |
| AC-8, 9, 10 and AC-19, 20 | 1 | 1 |  |
| AC-4, 5 and AC-3 | 1 |  | 1 |
| AC-6, 7 | 1 |  | 1 |

**Table S3. Target extractable nuclear antigen antibody in COVID-19 patients**

| Target ENA | All | Severe illness | Non-severe illness |
| --- | --- | --- | --- |
| Ro-52 | 15 | 9 | 6 |
| CB | 9 | 1 | 8 |
| M2 | 6 | 4 | 2 |
| Scl-70 | 4 | 3 | 1 |
| CB, RO-52 | 2 | 2 |  |
| M2, CB | 2 | 1 | 1 |
| PCNA | 2 | 2 |  |
| Ro-52, SSA | 2 | 2 |  |
| SSA | 2 | 1 | 1 |
| U1-RNP | 2 |  | 2 |
| CB, DNA | 1 | 1 |  |
| Hi | 1 |  | 1 |
| Jo-1 | 1 | 1 |  |
| M2, Ro-52 | 1 |  | 1 |
| PM-SCL | 1 | 1 |  |
| RIB | 1 | 1 |  |
| Ro-52, CENP-B | 1 |  | 1 |
| Ro-52, CENP-B, Pm-scl | 1 | 1 |  |
| Ro-52, SRP | 1 |  | 1 |
| Sm, U1-RNP | 1 | 1 |  |
| SSA, Ro-52 | 1 |  | 1 |
| SSA, Ro-52, SSB | 1 | 1 |  |
| SSB | 1 |  | 1 |
| U1-RNP, Hi, SSA, Ro-52 | 1 | 1 |  |
| U1-RNP, Sm, Hi | 1 |  | 1 |
